# Genetic characterization of atypical porcine pestivirus from neonatal piglets with congenital tremor in Hubei province, China

**DOI:** 10.1186/s12985-022-01780-8

**Published:** 2022-03-24

**Authors:** Xujiao Ren, Ping Qian, Zihui Hu, Huanchun Chen, Xiangmin Li

**Affiliations:** 1 State Key Laboratory of Agricultural Microbiology, Huazhong Agricultural University, Wuhan, 430070 Hubei China; 2 Laboratory of Animal Virology, College of Veterinary Medicine, Huazhong Agricultural University, Wuhan, 430070 Hubei China; 3 Key Laboratory of Preventive Veterinary Medicine in Hubei Province, The Cooperative Innovation Center for Sustainable Pig Production, Wuhan, 430070 Hubei China

**Keywords:** Atypical porcine pestivirus, APPV, Congenital tremor, Piglets, Hubei, Phylogenetic analysis

## Abstract

**Background:**

Atypical porcine pestivirus (APPV) is a single-stranded RNA virus with high genetic variation that causes congenital tremor (CT) in newborn piglets, belonging to the genus *Pestivirus* of the family *Flaviviridae*. Increasing cases of APPV infection in China in the past few years would pose severe challenges to the development of pig production. In view of the high genetic variability of APPV, the genetic characteristics of APPV in Hubei province was determined.

**Methods:**

52 tissue samples from 8 CT-affected newborn piglets were collected at two different periods in the same pig farm in Hubei province. Viral nucleic acid was extracted to detect pathogens that can cause CT in piglets or other common clinical pathogens by RT-PCR. Haematoxylin and eosin (HE) staining, immunohistochemical (IHC) analysis, and qRT-PCR were performed to observe histopathological changes and histological distribution, and detect the viral load of APPV in CT-affected piglets. The full-length genome of APPV was obtained and sequence analysis was conducted to determine the phylogenetic relationship.

**Results:**

Histopathological observation and histological distribution analysis showed that the histological lesions and distribution of APPV were mainly in central nervous system (CNS) tissues and immune tissues. Viral load analysis revealed that the viral copy number was higher in the cerebellum, submaxillary lymph nodes, tonsil, and serum than in other tissues. Phylogenetic analysis showed that CH-HB2020 and CH-HB2021 belonged to Clade I.3, and is most closely related to APPV_CH-GX2016. Sequence alignment based on APPV encoding sequences (CDS) showed that the nucleotide identities of CH-HB2020 or CH-HB2021 with Clade I, Clade II, and Clade III strains were 83.5–98.6%, 83.1–83.5%, and 81.1–81.4%, respectively, while the amino acid identities were 91.9–99.2%, 91.2–95.3%, and 90.77–91.4%, respectively. No recombination event was observed in CH-HB2020 or CH-HB2021 strains.

**Conclusions:**

These findings enhance our understanding of the pathogenesis of APPV and may provide potential molecular evidence for its prevalence and transmission.

**Supplementary Information:**

The online version contains supplementary material available at 10.1186/s12985-022-01780-8.

## Introduction

Atypical porcine pestivirus (APPV) is a member of the genus *Pestivirus* in the family *Flaviviridae* [1]. The genus *Pestivirus* also covers several recognized viruses, including bovine viral diarrhea virus-1 (BVDV-1), bovine viral diarrhea virus-2 (BVDV-2), border disease virus (BDV), and classical swine fever virus (CSFV). These viral species were redesignated as *Pestivirus* A (BVDV-1), *Pestivirus* B (BVDV-2), *Pestivirus* C (CSFV), and *Pestivirus* D (BDV), while the remaining newly discovered pestiviruses in recent years were renamed as *Pestivirus* E-K, of which APPV belongs to *Pestivirus* K [2]. The APPV genome is a single-stranded positive-sense RNA with a length of approximately 11–12 kb, containing a large open reading frame (ORF) flanked by 5’ and 3’ untranslated regions (UTRs). The ORF of APPV consists of 3635 amino acids (aa), which encodes four structural proteins (C, E^rns^, E1, and E2) and eight non-structural proteins (N^pro^, P7, NS2, NS3, NS4A, NS4B, NS5A, and NS5B) [1, 3–6].

APPV was first discovered in the United States in 2015. Using metagenomic sequencing, a novel pestivirus was identified in swine serum, sharing 68% nucleotide identity with Rhinolophus affinis pestivirus (RaPV) and 25–28% nucleotide identity to the other pestiviruses [1]. Recent studies have shown that the newly emerging virus was associated with CT type A-II in piglets [7, 8], which was characterized by rhythmic tremor in the limbs and head, complicated by ataxia. In recent years, infections with APPV have been reported in the Netherlands, Germany, China, Austria, Spain, Brazil, Canada, Hungary, Switzerland, Italy, and Japan [7–15], suggesting widespread distribution of this emerging virus worldwide.

So far, although numerous studies have been carried out on the epidemiology and genetic evolution of APPV in the past few years, there are relatively few reports on its pathogenesis, immune response, transmission route, and vaccine design. In the present study, various samples from 8 CT-affected newborn piglets at two different periods in the same pig farm in Hubei province were collected to detect APPV. The clinical and pathological features of CT in neonatal piglets were provided to better understand the pathogenesis of APPV. Two novel APPV isolates were identified and the complete genome sequences were characterized to investigate the genetic diversity and evolutionary relationships with other available APPV genomes. The results enhance our understanding of the pathogenesis of APPV and may provide potential evidence for its prevalence and transmission.

## Materials and methods

### Ethics statement

This study was approved by the Animal Ethical and Welfare Committee of the College of Veterinary Medicine, Huazhong Agricultural University, Hubei, China.

### Collection of CT clinical samples

52 tissue samples including heart, liver, spleen, lung, kidney, cerebrum, cerebellum, brainstem, spinal cord, submaxillary lymph nodes, tonsil, and serum from 8 CT-affected newborn piglets were collected at two different periods (18 November 2020 and 6 February 2021) in the same pig farm in Hubei province. Part of the tissue samples were fixed with 4% paraformaldehyde solution (Biosharp life sciences, Beijing Labgic Technology Co., Ltd., Beijing, China) for histopathological analysis, and the other samples were stored at – 80 °C until use.

### Viral nucleic acid extraction and pathogen detection

Tissue samples were homogenized and diluted with sterile PBS, followed by centrifugation at 6000 × *g* for 10 min at 4 °C to remove residual tissue debris. Viral nucleic acid was extracted using TRIpure Reagent (Aidlab, Beijing, China) or E.Z.N.A.^®^ Viral DNA Kit (Omega Bio-tek, Georgia, USA) in accordance with the manufacturer’s protocol.

A series of primers (Additional file 3: Table S1) were designed to detect pathogens that can cause CT in piglets or other common clinical pathogens, including APPV, CSFV, lateral-shaking inducing neurodegenerative agent (Linda) virus, porcine teschovirus (PTV), Japanese encephalitis virus (JEV), Seneca Valley virus (SVV), porcine sapelovirus (PSV), foot and mouth disease virus (FMDV), porcine reproductive and respiratory syndrome virus (PRRSV), porcine circovirus type 2 (PCV2), porcine circovirus type 3 (PCV3), and porcine pseudorabies virus (PRV) using methods as previously described [3, 16–19].

### Histopathological and immunohistochemical analysis

To observe histopathological changes and histological distribution of CT-affected piglets, haematoxylin and eosin (HE) staining and immunohistochemical (IHC) analysis were performed, as previously described [20]. Briefly, the fixed tissue samples were embedded in paraffin and cut into serial sections using a manual rotary microtome (HistoCore BIOCUT, Leica Biosystems, Shanghai, China). One part of sections were used for HE staining, and the others were subjected to IHC analysis with a monoclonal antibody (mAb) against APPV E2 [21].

### APPV isolation in cell culture

For virus isolation, APPV-positive serum was filtered using 0.22 µm filters, and then inoculated into PK-15 cells or SK6 cells in a 6-well plate (37 °C, 5% CO_2_). At 72 h post-infection, the cell supernatants were harvested and then inoculated into fresh cells. After successive generations, viral RNA was extracted for RT-PCR to detect APPV genome.

An indirect immunofluorescence assay (IFA) was performed to detect the expression of viral protein, as previously described [22]. Briefly, the infected cells were washed with phosphate buffer containing Tween 20 (PBST) three times, and then fixed with 4% paraformaldehyde solution for 30 min at room temperature (RT), permeabilized with 0.5% Triton X-100 (BioFroxx, Einhausen, Germany) for 20 min at − 20 °C, and further blocked with 5% BSA (BioFroxx, Einhausen, Germany) for 1 h at RT. The blocked cells were incubated with APPV E2 mAb (1:1000 dilution in PBS) [21] for 2 h at 37 °C. Subsequently, the cells were washed with PBST three times and incubated with Alexa Fluor 488 goat anti-mouse antibody (1:1000 dilution in PBS) (ThermoFisher, Waltham, MA, USA) for 1 h at 37 °C. After intensive washing, the cells were analyzed under a fluorescence microscope (Ti-U-Nikon, Tokyo, Japan) with a video documentation system.

### Viral load analysis

To detect the viral load of APPV in CT-affected piglets, quantitative reverse transcription-PCR (qRT-PCR) was carried out in a ViiA™ 7 Real‐Time PCR System (Applied Biosytems, Grand Island, NY, USA) with BlasTaq™ 2X qPCR MasterMix (abm, Richmond, BC, Canada), according to the manufacturer’s instructions. The pEASY-Blunt-APPV E2 plasmid [23] was used as the standard plasmid to draw a standard curve, and the copy number of APPV was calculated using the standard curve.

### Amplification of APPV genomes and phylogenetic analysis

To obtain the full-length genome of APPV, RT-PCR was performed using the primers listed in Additional file 4: Table S2 [24]. Viral RNA was extracted as described above, then cDNA was synthesized following the instruction of HiScript^®^ II Q RT SuperMix for qPCR (+ gDNA wiper) Kit (Vazyme Biotech Co., Ltd., Nanjing, China). Six separate fragments were amplified using PrimeSTAR^®^ HS DNA Polymerase (TaKaRa, Dalian, China). The RT-PCR procedures were as follows: 98 °C for 5 min, 30 cycles of denaturation at 98 °C for 10 s, annealing at 55 °C for 15 s, extension at 72 °C for 1 min, and a final step of 72 °C for 10 min. After purification with a TIANgel Midi Purification Kit (TIANGEN BIOTECH Co., Ltd, Beijing, China), the purified DNA was cloned into pMD18-T vector (TaKaRa, Dalian, China). Afterwards, the positive clones were sequenced by Sanger sequencing technology (Tsingke Biotechnology Co., Ltd., Wuhan, China). The sequences were obtained from three replicates to confirm the fidelity. The full-length genome sequence was assembled in Lasergene SeqMan 7.1.0 software (DNASTAR, Madison, WI, USA), and then deposited in GenBank.

A total of 71 reference sequences of APPV full-length genomes were downloaded from GenBank (accessed on 17 July 2021) for analysis. Phylogenetic analysis based on encoding sequences (CDS) of the whole genome and five partial genes of APPV (E2, E^rns^, N^pro^, NS3, and NS5B) were conducted in MEGA11 using the Maximum likelihood method with bootstrap analysis of 1000 replicates [25]. A rat pestivirus isolate (GenBank accession no.: NC_025677) from USA was used as an outgroup.

### Sequence alignment and recombination analysis

Nucleotide and amino acid sequences were aligned using Lasergene Megalign 7.1.0 software (DNASTAR, Madison, WI, USA). Potential recombination events were identified using Recombination Detection Program version 4 (RDP4) and then examined using SimPlot version 3.5.1 and Bootscan analysis [26].

## Results

### Description of CT clinical cases and detection of pathogens

In 18 November 2020 and 6 February 2021, a total of 8 CT-affected piglets were observed from the first parity in the same pig farm in Hubei province. CT-affected piglets exhibited typical clinical symptoms, including paroxysmal spasms and difficulty standing (Additional file 1: Video S1 and Additional file 2: Video S2).

**Fig. 1:**
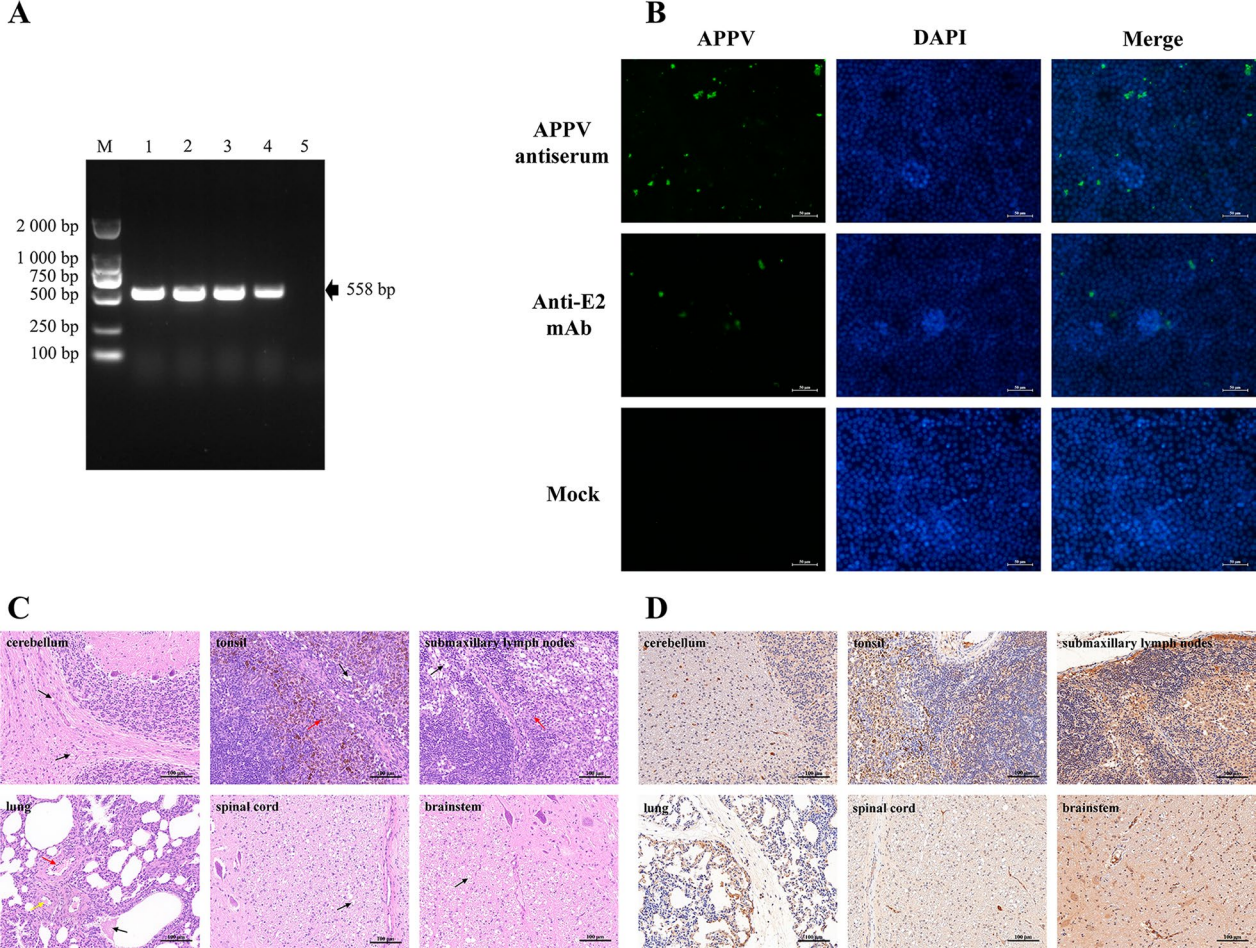
Identification of APPV in CT-affected piglets. **A** Detection of APPV in CT-positive samples. Four cerebellums were chosen to detect the APPV genome based on partial NS3 gene (Lane 1–4). ddH_2_O was used as a negative control (Lane 5). **B** Detection of the viral protein expression in PK-15 cells. The viral protein was detected in PK-15 cells inoculated with APPV-positive serum or APPV E2 mAb, mock-infected cells (PK-15) were used as the negative control. **C** Histological lesions in a CT-affected piglet. **D** Immunohistochemical detection in a CT-affected piglet

Cerebellum samples were chosen to detect the APPV genome based on partial NS3 gene, and all samples were positive for APPV (Fig. 1A). In addition, RT-PCR was performed to detect whether there were other viruses co-infected with APPV. However, no other pathogens were detected (data not shown).

### APPV isolation in cell culture

The initial attempt to isolate APPV was conducted in PK-15 cells or SK6 cells. Unfortunately, we could not detect the viral genome by RT-PCR after 6 passages. IFA results showed that the propagation efficiency of APPV in PK-15 cells was low, as indicated by the small amount of viral protein was detected (Fig. 1B).

Porcine complement regulatory protein CD46 is a major receptor for APPV entry [27]. Therefore, we tried to construct a PK-15 cell line over-expressing CD46 to improve the proliferation efficiency of APPV, but as in PK-15 cells, the viral genome was lost after 6 passages (data not shown).

### Histopathological observation and histological distribution

To observe histopathological changes of CT-affected piglets, HE staining was conducted. As shown in Fig. 1C, few vacuoles were found in the cerebellar white matter. In the tonsil, vacuoles and hemosiderosis were present in the lymphocytes. Severe edema of lymphocytes was observed in the submaxillary lymph nodes. In the lung, inflammatory cell infiltration and cellulose exudation were found in the alveolar capillaries or alveolar cavities. Hypomyelination and vacuoles were evident in the white matter of the spinal cord, and the brainstem exhibited severe edema.

For histological distribution analysis, immunohistochemical was performed (Fig. 1D). In the cerebellum, the white matter and granular layer showed moderate positive staining. In the tonsil, strong positive staining was observed in the diffuse lymphoid tissue, and moderate positive staining was present in the lymphoid nodule. In the submaxillary lymph nodes, the cortex and capsule showed strong positive staining. The epithelial cells of the pulmonary alveolus exhibited moderate positive staining. The white matter of the spinal cord was weak positive staining. The matrix and nerve fibres were stained moderately in the brainstem.

### Viral load analysis

**Fig. 2:**
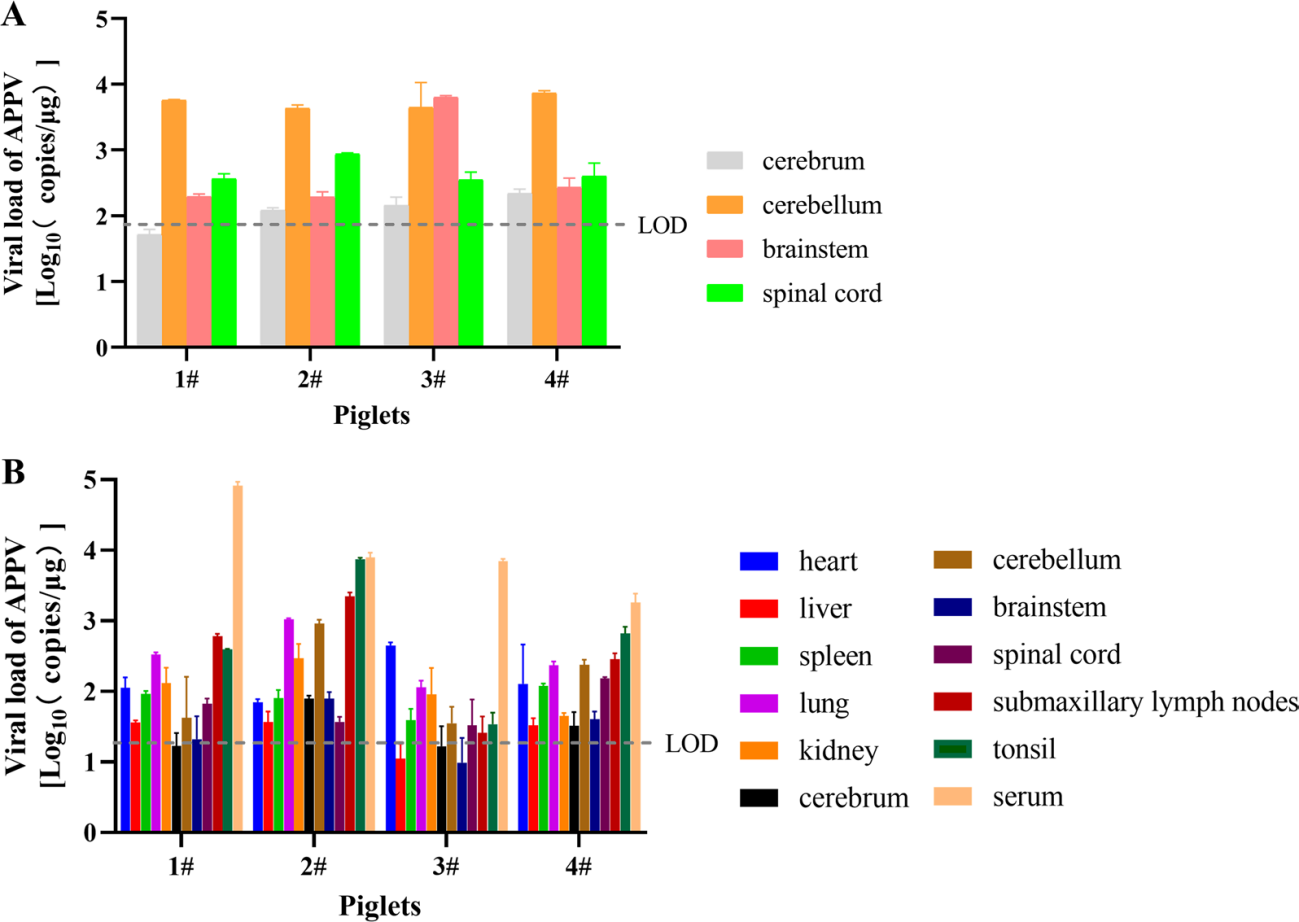
Viral load of APPV in CT-affected piglets. **A** Viral load of APPV in the central nervous system of CT-affected piglets. **B** Viral load of APPV in various tissues of CT-affected piglets. Various tissues (heart, liver, spleen, lung, kidney, cerebrum, cerebellum, brainstem, spinal cord, submaxillary lymph nodes, tonsil, and serum) from CT-affected piglets were tested to determine the copy number of APPV by absolute qRT-PCR. Data are represented as the mean ± SD. LOD, limit of detection

For the first batch of samples (collected in 18 November 2020), only the central nervous system (CNS) was analyzed, and the results showed that the highest copy number of APPV was found in the cerebellum and the lowest copy number was found in the cerebrum (Fig. 2A). To fully characterize the viral load of APPV in various tissues, we expanded the detection range in the second batch of samples (6 February 2021). As shown in Fig. 2B, the copy number of APPV in serum was the highest among all tested samples, suggesting that serum can be used to replace other tissues in clinical sampling and detection of APPV or virus isolation to reduce costs and improve efficiency.

### Phylogenetic analysis

**Fig. 3:**
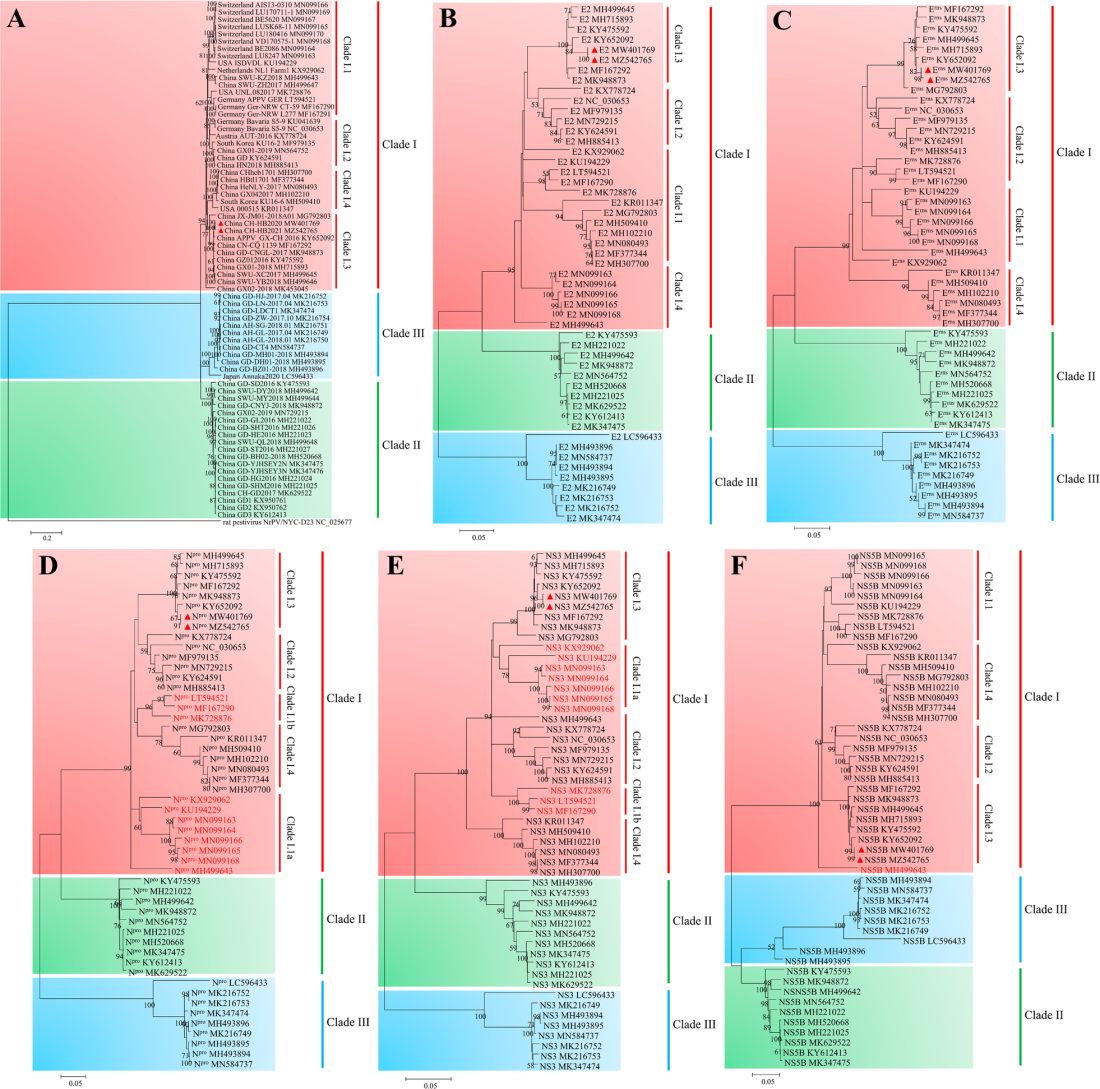
Phylogenetic analysis of all available APPV strains. Phylogenetic analysis based on encoding sequences of the whole genome (**A**), E2 (**B**), E^rns^ (**C**), N^pro^ (**D**), NS3 (**E**), and NS5B genes (**F**) were conducted in MEGA11 using the Maximum likelihood method and GTR + G + I (for full-length CDS, N^pro^, NS3, and NS5B) or TN93 + G + I (for E2 and E^rns^) substitution model. Bootstrap values were calculated for 1000 replicates, only values > 50% are indicated. A rat pestivirus isolate (NC_025677) from USA was used as an outgroup. ▲ indicates the sequence from the virus described in this study (color figure online)

Two APPV strains were identified, and submitted to GenBank under accession numbers MW401769 and MZ542765. Phylogenetic analysis based on CDS of the whole genome revealed that APPV genotypes clustered into three large clades, of which Clade II and III were almost exclusively Chinese strains, except for a Japanese strain (GenBank accession no.: LC596433) reported recently belonged to Clade III. Clade I was composed of strains from three continents, including Switzerland, the Netherlands, Germany, Austria, the United States, South Korea, and China, indicating a wide geographic distribution of APPV. The CH-HB2020 and CH-HB2021 strains (GenBank accession no.: MW401769 and MZ542765) in this study were located in the Clade I.3 branch (Fig. 3A). Phylogenetic tree based on E2, E^rns^, and NS5B genes was consistent with that based on full-length CDS (Fig. 3B–D), suggesting that E2, E^rns^, and NS5B genes could be used for APPV genotyping instead of full-length CDS.

### Sequence identities and recombination analysis

**Fig. 4:**
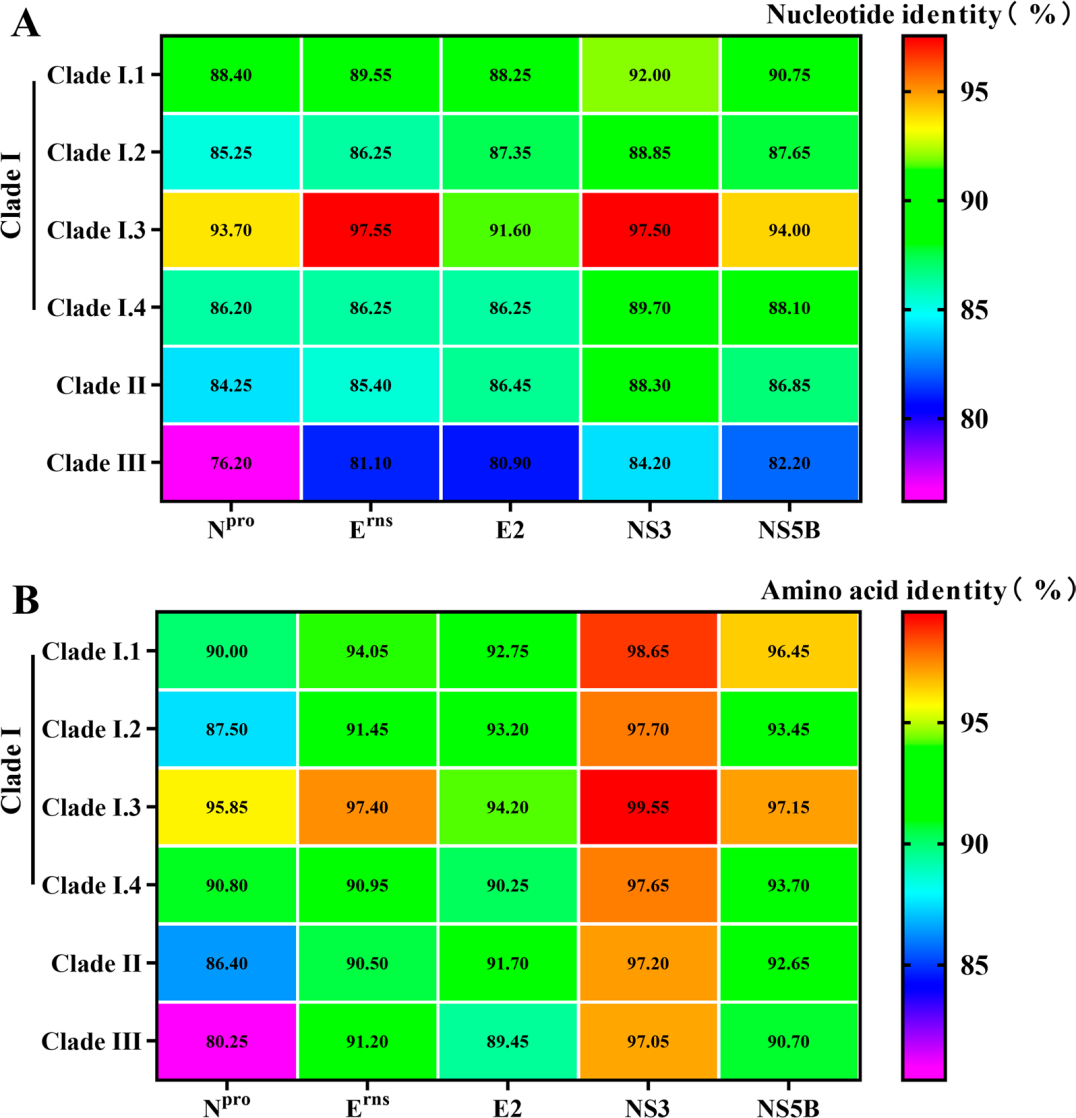
Nucleotide (**A**) and amino acid sequence (**B**) identities of the N^pro^, E^rns^, E2, NS3, and NS5B genes between CH-HB2020/2021 and other APPV isolates. Nucleotide and amino acid sequences were aligned using Lasergene Megalign 7.1.0 software, and then visualized with heat map in GraphPad Prism 8.0 software

Comparison of nucleotide and amino acid sequences based on APPV CDS showed that the nucleotide identities of CH-HB2020 or CH-HB2021 with Clade I, Clade II, and Clade III strains were 83.5–98.6%, 83.1–83.5%, and 81.1–81.4%, respectively (Table 1), while the amino acid identities were 91.9–99.2%, 91.2–95.3%, and 90.77–91.4%, respectively (Table 2). Homology analysis based on N^pro^, E^rns^, E2, NS3, and NS5B genes among different strains indicated that the nucleotide and amino acid sequences of N^pro^ were the most different from other strains, while NS3 was relatively conservative (Fig. 4).

**Table 1:** Nucleotide identities (%) of the complete CDS between CH-HB2020/2021 and other APPV isolates

Nucleotide identities (%)	HB2020/2021	Clade I				Clade II	Clade III
		Clade I.1	Clade I.2	Clade I.3	Clade I.4		
HB2020/2021	–	89.7–90.6	83.5–90.8	88.7–98.6	87.3–87.9	83.1–83.5	81.1–81.4

**Table 2:** Amino acid sequence identities (%) of the complete CDS between CH-HB2020/2021 and other APPV isolates

Amino acid sequence identities (%)	HB2020/2021	Clade I				Clade II	Clade III
		Clade I.1	Clade I.2	Clade I.3	Clade I.4		
HB2020/2021	–	94.8–96.1	91.9–95.7	95.1–99.2	94.1–94.4	91.2–95.3	90.7–91.4

Since CH-HB2020 or CH-HB2021 and APPV_CH-GX2016 strains (GenBank accession no.: KY652092) were closely related (Fig. 3A), the amino acid differences between them were compared in order to further explore the evolutionary relationship between them. Additionally, the amino acid differences between CH-HB2021 and CH-HB2020 strains in the same field were also compared. As shown in Table 3, compared with APPV_CH-GX2016 strain, the total mutation frequency of amino acids in CH-HB2021 strain was 0.99% (36/3635), among which E2 gene had the highest mutation frequency (2.90%, 7/241). Compared with CH-HB2020 strain, CH-HB2021 had only 6 amino acid mutations. In pestiviruses, E2 protein is the main protective antigen that induces the production of neutralizing antibodies [28], suggesting that the mutation of E2 gene may be related to the antiviral response mechanism of the virus escaping the host.

**Table 3:** Differential amino acid residues of the CDS between CH-HB2021 (MZ542765) strain and other isolates

GenBank no./strain	Gene	Differential amino acid sites	Replacement rate	Total
KY652092/APPV_GX-CH 2016	N^pro^	G105S, L145M, R162G	3/180 (1.67%)	36/3635 (0.99%)
	C	V75I, T107I, Y119H	3/111 (2.70%)	
	E^rns^	A105G, Y119H, N159S, A169V	4/210 (1.90%)	
	E1	A2V	1/199 (0.50%)	
	E2	E6Q, K18R, G35R, N47K, G52N, I67S, R118G	7/241 (2.90%)	
	NS2	E127K	1/314 (0.32%)	
	NS4B	D14T, A16V, V30A, S72F	4/339 (1.18%)	
	NS5A	R52Q, V114I, T115I, D407N, T410M, A433T	6/472 (1.27%)	
	NS5B	S5N, D85N, I142V, S189N, I445M, P497L, T728I	7/751 (0.93%)	
MW401769/CH-HB2020	E^rns^	A103G	1/210 (0.48%)	6/3635 (0.17%)
	NS2	E127K	1/314 (0.32%)	
	NS4B	D14F, A20V, S72F	3/339 (0.88%)	
	NS5B	P497L	1/751 (0.13%)	

**Fig. 5:**
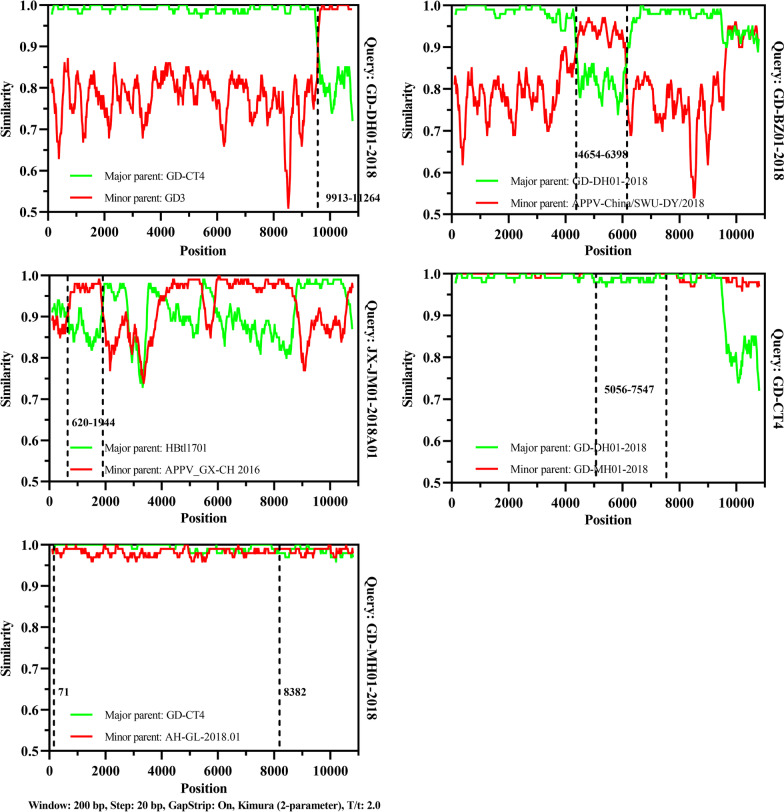
Recombination analysis of all available APPV strains based on the encoding sequences. Potential recombination events were identified using Recombination Detection Program version 4 (RDP4) and then examined using SimPlot version 3.5.1 and Bootscan analysis. Additional details were represented in Additional file 5: Table S3

To further explore the genetic evolution of APPV, potential recombination events were identified using Recombination Detection Program version 4 (RDP4) and then examined using SimPlot version 3.5.1 and Bootscan analysis. The results showed that among all available APPV strains, a total of 5 APPV strains (GD-DH01-2018, GD-BZ01-2018, JX-JM01-2018A01, GD-CT4, and GD-MH01-2018) were found to have potential genetic recombination events (Fig. 5 and Additional file 5: Table S3), while no genetic recombination occurred in CH-HB2020 or CH-HB2021 strains in this study.

Overall, these results suggest that APPV is a virus with high genetic variation, which poses a challenge to the prevention and control of CT and epidemiological surveillance in piglets caused by APPV.

## Discussion

Congenital tremor (CT) of piglets has developed into a global disease since it was first reported a century ago [29]. CT-affected piglets regularly exhibit varying degrees of muscle tremor and hind leg syndrome within hours of birth. Sometimes there is rhythmic tremor in the head and instability in the limbs, further complicated by ataxia. Such symptoms lead to difficulty in suckling, followed by starvation due to insufficient colostrum intake and death, with a mortality rate of 10–30%, bringing great economic losses to the pig industry [30]. According to pathological features and etiology, CT can be divided into different subtypes (types A I-V and type B). Currently, accumulating evidence indicates that CT type A-II is associated with APPV [7, 8], drawing considerable attention to this virus.

In this study, APPV was detected from neonatal piglets with congenital tremor in Hubei province. Histopathological findings of affected piglets were mainly characterized by increased vacuoles in the white matter of the cerebellum and brainstem, hypomyelination of the spinal cord, which was consistent with previous work [13]. The histological lesions of the CNS may be responsible for neurological signs such as tremor or ataxia in piglets. Additionally, the peripheral lymphoid organs including tonsils and lymph nodes also showed histological changes, suggesting that APPV may have immunosuppressive effects on the host [31]. Undoubtedly, the mechanism of CNS or immune organs lesions caused by APPV infection will be verified in future studies. The low proliferation efficiency of APPV in cell cultures severely hampers the intensive study of the virus. We tried to isolate APPV in PK-15 cells or SK6 cells, and PK-15-CD46 cells, a cell line over-expressing the major receptor for APPV entry, unfortunately, we could not detect the viral genome by RT-PCR after 6 passages. It is exciting that a recent study showed that APPV could effectively propagated in embryonic porcine kidney epithelial cells (SPEV cells) [32, 33], which will facilitate the development of APPV research.

**Fig. 6:**
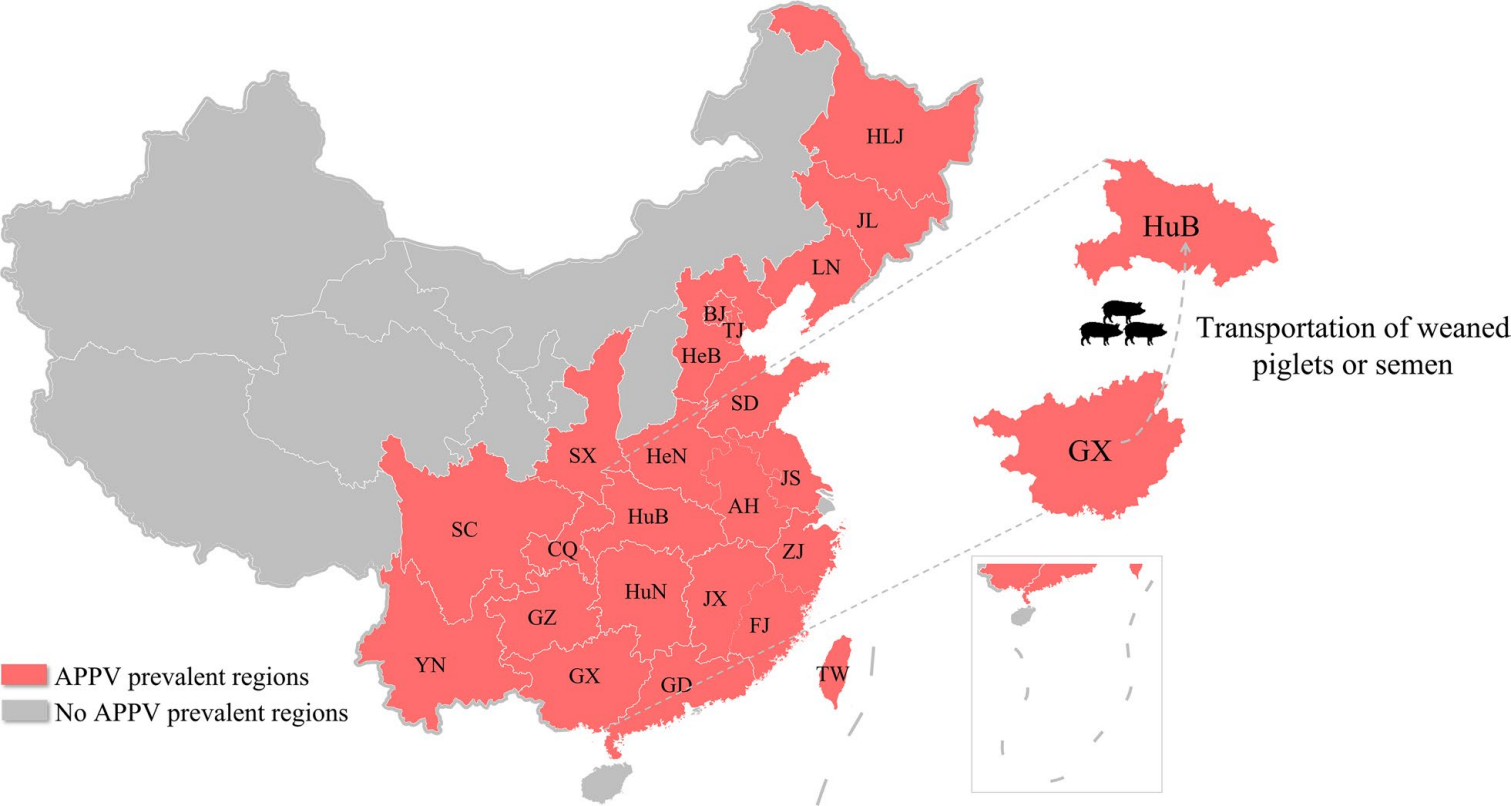
Geographical distribution of APPV in China. APPV prevalent regions were shown in pink. The transregional transmission route of APPV proposed in this study was also shown

In 2016, two separate research groups detected APPV infection in swine herds almost simultaneously for the first time in China. Nucleotide identities between available APPV sequences were 83.1–83.5% [14] and 88.3–93.5% [34], respectively, showing that APPV is highly diverse in China. Previous study has shown that APPV clustered into three clades, including a novel branch, and thus indicating the emergence of a new genotype and complex APPV dissemination in China [35]. Our analyses showed that CH-HB2020 and CH-HB2021 belonged to Clade I.3, and is most closely related to APPV_CH-GX2016. Additionally, CH-HB2020 and CH-HB2021 were isolated from the same pig farm, suggesting that persistent infection (PI) of APPV can occur within a pig farm [7]. Notably, we learned that the pig farm where APPV_CH-GX2016 strain was isolated belongs to the same breeding enterprise as the pig farm where CT occurred in this study. And these farms received weaned piglets or semen used for artificial insemination from the same sow farm or breeding farm. Therefore, we speculated that APPV may be transmitted across regions through weaned piglets or semen transportation (Fig. 6). However, more convincing evidence needs to be provided in the future study. APPV is capable of vertical and horizontal transmission, which has been verified in many studies [7, 13, 36, 37]. Therefore, to block the dissemination of APPV, strenuous efforts should be made to acclimatize reserved gilts and strengthen the quarantine of commercial pigs and their products, especially the quarantine of boar semen used for artificial insemination. Furthermore, homology analysis revealed that N^pro^ had the highest genetic variability among all available strains, while E2 gene mutation frequency was the highest between CH-HB2021 and APPV_CH-GX2016 strain. In pestiviruses, N^pro^ protein plays a critical role in inhibiting interferon (IFN) production and thus evading the host innate immune response [31, 38–41]. E2 protein is the main protective antigen that induces a strong immune response [21, 23, 28, 42], and therefore may be subjected to greater selection pressure. Thus, the high variability of N^pro^ and E2 genes may be one of the reasons for persistent infection and co-infection of APPV [13, 43, 44].

High genetic variability of APPV genome was attributed to the point mutation and homologous recombination [26, 45]. Our results showed that among all available APPV strains, a total of 5 APPV strains within Clade I and Clade III were found to have potential genetic recombination events, while no recombination event occurred in CH-HB2020 or CH-HB2021 strains in this study. Increasing cases of APPV infection in China in the past few years would pose severe challenges to the development of pig production. Referring to the high genetic variability of APPV, continuous research is needed to determine the prevalence of the virus.

## Conclusions

In conclusion, in the present study, we described a CT case of newborn piglets caused by APPV infection in Hubei province. The histological lesions of the CNS may be responsible for neurological signs such as tremor or ataxia in piglets. Viral load analysis revealed that serum could be the first choice for clinical sample collection. Phylogenetic analysis showed that CH-HB2020 and CH-HB2021 belonged to Clade I.3, and is most closely related to APPV_CH-GX2016. Furthermore, phylogenetic tree based on E2, E^rns^, and NS5B genes was consistent with that based on full-length CDS, suggesting that E2, E^rns^, and NS5B genes could be used for APPV genotyping instead of full-length CDS. The high variability of N^pro^ and E2 genes may be one of the reasons for persistent infection and co-infection of APPV. The results provide potential molecular evidence for transregional transmission of APPV through transportation.

## Supplementary Information

**Additional file 1: Video S1.** CT-affected piglets exhibited typical clinical symptoms, including paroxysmal spasms and difficulty standing.

**Additional file 2: Video S2.** CT-affected piglets exhibited typical clinical symptoms, including paroxysmal spasms and difficulty standing.

**Additional file 3: Table S1.** Primers used for co-infection detection in this study.

**Additional file 4: Table S2.** Primers used for APPV genome amplification in this study.

**Additional file 5: Table S3.** Recombination events detection and analysis of all available APPV strains.

**Additional file 6: Sanger sequencing peak maps.** The base quality values were shown in the Sanger sequencing peak maps.

## Data Availability

The data that support the findings of this study are available from the corresponding author upon reasonable request.

## Abbreviations

APPV: Atypical porcine pestivirus
CT: Congenital tremor
HE: Haematoxylin and eosin
IHC: Immunohistochemical
CNS: Central nervous system
CDS: Encoding sequences
ORF: Open reading frame
UTRs: Untranslated regions
RT‑PCR: Reverse‑transcription polymerase chain reaction
qRT-PCR: Quantitative reverse‑transcription polymerase chain reaction
BVDV-1: Bovine viral diarrhea virus-1
BVDV-2: Bovine viral diarrhea virus-2
BDV: Border disease virus
CSFV: Classical swine fever virus
PTV: Porcine teschovirus
JEV: Japanese encephalitis virus
SVV: Seneca Valley virus
PSV: Porcine sapelovirus
FMDV: Foot and mouth disease virus
PRRSV: Porcine reproductive and respiratory syndrome virus
PCV2: Porcine circovirus type 2
PCV3: Porcine circovirus type 3
PRV: Porcine pseudorabies virus
