# Supplementary material for: Genetic characterization of atypical porcine pestivirus from neonatal piglets with congenital tremor in Hubei province, China
Source: Virol J. 2022 Mar 24;19:51. doi: 10.1186/s12985-022-01780-8 (PMC8944037; doi:10.1186/s12985-022-01780-8)
Supplement: Supplementary file 3 — Additional file 3: Table S1. Primers used for co-infection detection in this study. [file 12985_2022_1780_MOESM3_ESM.docx]

**Table S1** Primers used for co-infection detection in this study

| **Primers** | **Sequence (5’ to 3’)** | **Length (bp)** | **Target** | **Reference** |
| --- | --- | --- | --- | --- |
| APPV-D-F | GACCCCAGTCCACTCAACGA | 558 | Atypical porcine pestivirus, NS3 | NA |
| APPV-D-R | AACTCTCCCCTCCGCATATC |  |  |  |
| CSFV-D-F | TAGGGTGGACGGGTGTCATAGAGT | 536 | Classical swine fever virus, E2 | [17] |
| CSFV-D-R | AAGCATATATTGCTGGAAGTAGCT |  |  |  |
| LindaV-D-F | ATCCAAGCATAGCATTCAACC | 348 | Lateral-shaking inducing neurodegenerative agent, NS3 | [3] |
| LindaV-D -R | TGTCCAAATAATCCTCGCCT |  |  |  |
| PTV-D-F | TGTTGTGTTTAAACACAGAAAT | 306 | Porcine teschovirus, 5’UTR | [19] |
| PTV-D-R | TTCAACTGACTATACAAAGTAC |  |  |  |
| JEV-D-F | GAGCTTGTTGGACGGCAGAG | 181 | Japanese encephalitis virus, C | NA |
| JEV-D-R | CACGGCGTCGATGAGTGTTC |  |  |  |
| SVV-D-F | GTTGTAACTACAAGATTTAGC | 365 | Seneca Valley virus, 5’UTR | NA |
| SVV-D-R | CAGGCAGTATCCAAGGCACG |  |  |  |
| PSV-D-F | TGCCAGGGAGAGTTACAAAT | 494 | Porcine sapelovirus, VP3 | NA |
| PSV-D-R | GGGCACTACTAAGGCTGTTT |  |  |  |
| FMDV-D-F | TCACACAGTTTTGCCCGTTTTT | 462 | Foot and mouth disease virus, 5’UTR | NA |
| FMDV-D-R | CAGTCCCCTTCTCAGATCCCG |  |  |  |
| PRRSV-D-F | TCCCAGTGCCAAGTGGACAT | 500 | Porcine reproductive and respiratory syndrome virus, GP2 | NA |
| PRRSV-D-R | CAGCGGAAACCAAAAACAGTAC |  |  |  |
| PCV2-D-F | TTTTATTGTTGGCGAGGAGG | 429 | Porcine circovirus type 2, ORF1 | NA |
| PCV2-D-R | GCAGCCCATTTGCTTTTACC |  |  |  |
| PCV3-D-F | AGGGAAAGCCCGAAACACAG | 395 | Porcine circovirus type 3, replication-associated protein | NA |
| PCV3-D-R | GCAGGCATCTTCTCCGCAAC |  |  |  |
| PRV-D-F | GCTGACGCTGACGACGGTCCCCT | 491 | Porcine pseudorabies virus, gD | NA |
| PRV-D-R | CGCCGAACTTGTACGTGCGGTGCT |  |  |  |

NA: Not available. These primers were designed in house.
