# Supplementary material for: Genetic characterization of atypical porcine pestivirus from neonatal piglets with congenital tremor in Hubei province, China
Source: Virol J. 2022 Mar 24;19:51. doi: 10.1186/s12985-022-01780-8 (PMC8944037; doi:10.1186/s12985-022-01780-8)
Supplement: Supplementary file 4 — Additional file 4: Table S2. Primers used for APPV genome amplification in this study. [file 12985_2022_1780_MOESM4_ESM.docx]

**Table S2** Primers used for APPV genome amplification in this study

| **Primers** | **Sequence (5’ to 3’)** | **Length (bp)** | **Primer location (nt)^a^** |
| --- | --- | --- | --- |
| S1-F | TGCTTTGATTGGCTGCATTA | 2210 | 7-2216 |
| S1-R | CTGGCTGGGAGTTCTATTGT |  |  |
| S2-F | GCACTTGTTTGGCTCAGGTA | 2394 | 2028-4421 |
| S2-R | TCTATCCCCAGGAAGGTCAT |  |  |
| S3-F | CTTATTTTAGGAGAGCTGGGTT | 1997 | 4273-6269 |
| S3-R | GCTTGTGGGTTGACATCTTTTAG |  |  |
| S4-F | GGGTCTTTATGATGTAGATGAA | 2283 | 6012-8294 |
| S4-R | CATACAATGTCCCTGATTTCTG |  |  |
| S5-F | AAGATCACGGATCAACTCACTA | 1681 | 8224-9904 |
| S5-R | TTGGCATGGCTGTTTCGTAGTA |  |  |
| S6-F | CTCTAAGTTCAGGCAATCAAT | 1855 | 9606-11460 |
| S6-R | TCATTCAAGTATTTACAACAACC |  |  |

a: Numbers correspond to positions within the strain APPV_CH-GX2016.
