# Supplementary material for: Genetic characterization of atypical porcine pestivirus from neonatal piglets with congenital tremor in Hubei province, China
Source: Virol J. 2022 Mar 24;19:51. doi: 10.1186/s12985-022-01780-8 (PMC8944037; doi:10.1186/s12985-022-01780-8)
Supplement: Supplementary file 5 — Additional file 5: Table S3. Recombination events detection and analysis of all available APPV strains. [file 12985_2022_1780_MOESM5_ESM.docx]

**Table S3** Recombination events detection and analysis of all available APPV strains

| Recombinant | Major parent | Minor parent | Breakpoint position in alignment | Methods (Av. *P*-Val) | | | | | | | Recombinant score |
| --- | --- | --- | --- | --- | --- | --- | --- | --- | --- | --- | --- |
|  |  |  |  | RDP | GENECONV | BootScan | MaxChi | Chimaera | SiScan | Phylpro |  |
| GD-DH01-2018 | GD-CT4 | GD3 | 9913-11264 | 9.18×10^-133^ | 9.54×10^-201^ | 1.35×10^-132^ | 1.32×10^-29^ | 1.65×10^-20^ | 4.90×10^-31^ | 2.13×10^-7^ | 0.75 |
| GD-BZ01-2018 | GD-DH01-2018 | APPV-China/SWU-DY/2018 | 4654-6398 | 1.76×10^-111^ | 1.47×10^-55^ | 6.01×10^-107^ | 2.61×10^-32^ | 1.23×10^-5^ | 5.65×10^-27^ | 2.03×10^-11^ | 0.70 |
|  | GD-CT4 | APPV-China/SWU-DY/2018 | 4156-4223 | -- | 6.04×10^-9^ | -- | -- | -- | -- | 4.05×10^-2^ | 0.69 |
|  | AH-SG-2018.01 | GD-CT4 | 1894-3410 | 1.60×10^-3^ | 8.59×10^-6^ | 1.99×10^-7^ | 4.49×10^-5^ | 4.63×10^-3^ | 1.63×10^-5^ | 2.56×10^-2^ | 0.65 |
|  | AH-GL-2017.04 | GD-ZW-2017.10 | 4250-7728 | 2.86×10^-3^ | 3.62×10^-3^ | 1.50×10^-4^ | 1.15×10^-4^ | 1.82×10^-4^ | 1.70×10^-7^ | 1.15×10^-2^ | 0.66 |
| JX-JM01-2018A01 | HBtl1701 | APPV/CNGL/2017 | 6166-9058 | 1.71×10^-57^ | 3.31×10^-54^ | 4.72×10^-55^ | 2.14×10^-31^ | 2.07×10^-30^ | 3.51×10^-46^ | 5.08×10^-12^ | 0.67 |
|  | KU16-6 | APPV-China/GD-SHT/2016 | 2938-3686 | 2.27×10^-30^ | 3.92×10^-13^ | 6.10×10^-31^ | 6.80×10^-15^ | 3.57×10^-16^ | 1.35×10^-13^ | 1.52×10^-11^ | 0.69 |
|  | HBtl1701 | APPV_GX-CH 2016 | 620-1944 | 9.00×10^-26^ | 1.69×10^-25^ | 8.97×10^-28^ | 4.41×10^-10^ | 8.27×10^-11^ | 2.53×10^-22^ | 5.08×10^-12^ | 0.72 |
|  | HBtl1701 | APPV/CNGL/2017 | 4449-5690 | 3.60×10^-37^ | 7.80×10^-35^ | 1.40×10^-38^ | 3.93×10^-18^ | 1.88×10^-18^ | 6.32×10^-21^ | 5.08×10^-12^ | 0.71 |
|  | APPV-China/HeNLY/2017 | APPV/CNGL/2017 | 10903-11258 | 2.31×10^-13^ | 2.34×10^-9^ | 1.65×10^-13^ | 1.12×10^-6^ | 2.95×10^-6^ | 1.69×10^-6^ | 1.02×10^-11^ | 0.71 |
|  | GD-LDCT1 | GD-HJ-2017.04 | 10638-2378 | 3.06×10^-2^ | 2.93×10^-4^ | 1.58×10^-11^ | 1.35×10^-7^ | 3.04×10^-5^ | -- | -- | 0.61 |
| GD-CT4 | GD-DH01-2018 | GD-MH01-2018 | 5056-7547 | 3.08×10^-4^ | 3.43×10^-2^ | 3.13×10^-4^ | 2.92×10^-4^ | -- | -- | 1.09×10^-3^ | 0.48 |
| GD-MH01-2018 | GD-CT4 | AH-GL-2018.01 | 8382-71 | -- | -- | -- | 1.17×10^-7^ | 2.02×10^-3^ | -- | -- | 0.53 |

--: not significant
